# Usefulness of pretransplant aortic arch calcification evaluation for kidney transplant outcome prediction in one year follow-up

**DOI:** 10.1080/0886022X.2018.1455588

**Published:** 2018-04-05

**Authors:** Agne Laucyte-Cibulskiene, Evelina Boreikaite, Gediminas Aucina, Migle Gudynaite, Ilona Rudminiene, Sigita Anisko, Loreta Vareikiene, Liutauras Gumbys, Dileta Valanciene, Ligita Ryliskyte, Kestutis Strupas, Laurynas Rimsevicius, Marius Miglinas

**Affiliations:** a Clinic of Gastroneterology, Nephrourology and Abdominal Surgery, Institute of Clinical Medicine, Faculty of Medicine, Vilnius University, Vilnius, Lithuania;; b Faculty of Medicine, Vilnius University, Vilnius, Lithuania;; c Centre of Nephrology, Vilnius University Hospital Santaros Clinics, Vilnius, Lithuania;; d Centre of Radiology and Nuclear Medicine, Vilnius University Hospital Santaros Clinics, Vilnius, Lithuania

**Keywords:** Kidney transplantation, aortic arch calcification, aortic stiffness, cardiovascular events, kidney graft function

## Abstract

Vascular calcification (VC) is linked to post-transplant cardiovascular events and hypercalcemia which may influence kidney graft function in the long term. We aimed to evaluate whether pretransplant aortic arch calcification (AoAC) can predict post-transplant cardiovascular or cerebrovascular events (CVEs), and to assess its association with post-transplant plasma calcium levels and renal function in one-year follow-up. Our single-center observational prospective study enrolled 37 kidney transplant recipients (KTR) without previous history of vascular events. Two radiologists evaluated pretransplant AoAC on chest X-ray as suggested by Ogawa et al. in 2009. Cohen’s kappa coefficient was 0.71. The mismatching results were repeatedly reviewed and resulted in consensus. Carotid-femoral (cfPWV) and carotid-radial pulse wave velocity (crPWV) was measured using applanation tonometry before and one year after transplantation. Patient clinical, biochemical data, and cardiovascular/CVE rate were monitored within 1 year. We found out that eGFR_1year_ correlated with eGFR_discharge_ and calcium based on hospital discharge data (*β* = 0.563, *p* = .004 and *β* = 51.360, *p* = .026, respectively). Multivariate linear regression revealed that donor age, donor gender, and recipient eGFR_discharge_ (*R*-squared 0.65, *p* = .002) better predict eGFR_1year_ than AoAC combined with recipient eGFR_discharge_ (*R*-squared 0.35, *p* = .006). During 1-year follow-up, four (10.81%) patients experienced cardiovascular events, which were predicted by PWV ratio (HR 7.549, *p* = .045), but not related to AoAC score (HR 1.044, *p* = .158). In conclusion, KTR without previous vascular events have quite low cardiovascular/CVE rate within 1-year follow-up. VC evaluated as AoAC on pretransplant chest X-ray together with recipient eGFR_discharge_ could be related to kidney function in one-year follow-up.

## Introduction

Kidney transplantation (KT) is the most effective treatment method for patients with end-stage renal disease (ESRD) that increases life expectancy [1], but these patients suffer from higher cardiovascular mortality than the general population [2,3]. More than half of ESRD patient deaths are caused by cardiovascular diseases [4]. Recent studies imply that vascular calcification (VC) is an important predictor of cardiovascular and all-cause mortality in chronic hemodialysis patients [5–9] and is detected for more than 50% predialysis patients and for 80–90% patients with ESRD [10,11]. As in ESRD patients, VC in KT recipients strongly predicts post-transplant cardiovascular outcomes [12].

Interrelationship between aortic calcification and aortic stiffness [13,14], a hallmark of vascular aging [15], have been shown in ESRD patients. Sekikawa et al. have observed that the association of aortic calcification with stiffness begins as early as the 40s in healthy individuals [16], also another study has proved progression of coronary artery calcification in ESRD patients undergoing dialysis at the 30s [17]. Results of previous studies suggest that aorta calcification is associated with decreased GFR in patients with stages 3–5 CKD [18,19]. Aortic smooth muscle cell calcification is known to be associated with excess phosphate influenced by lower urinary excretion in ERSD patients [20,21]. There are few studies which have evaluated post-transplant PWV in the context of VC [22–24].

The pathogenesis of VC could involve either the intima layer, linking to inflammation and atherosclerosis, or the media layer, causing vascular stiffness [25]. Vascular calcification is available to screen with a number of noninvasive imaging techniques. Even though, electron beam computer tomography and multi-detector row computer tomography are known as a gold standards for assessment of the extent of VC [26–29], they are lacking in cost-effectiveness and simplicity of technique routinely performed in clinical practice. Ogawa et al. proposed a simple scale combined with chest radiography for evaluation of aortic arch calcification (AoAC) [30], which has proved to be an efficient tool in screening of AoAC and identifying patients at the risk of cardiovascular events [31,32].

Many present prospective observational studies focus on evaluation of coronary artery calcification [12], but there are still very few studies that have examined the cost-efficient AoAC detection on chest X-ray in routine practice and its association with increased cardiovascular morbidity and mortality [24]. Additionally, there are no studies which evaluate pretransplant VC in the context of posttransplant allograft function. Safar et al. reported that first year after KT recipient eGFR decline was significantly linked to smoking and acute rejection and after nine years of follow up – to donor age and donor aortic stiffness [33]. We hypothesized that due to possible reabsorption of VC after kidney transplant there could be increase in serum calcium concentration which together with other factors causes compensatory polyuria and increase in eGFR. So, the aim of this study was to evaluate whether pretransplant AoAC grade or arterial stiffness can predict cardiovascular or cerebrovascular events (CVEs) and post-transplant renal graft function.

## Methods

### Study population and design

Single-center observational prospective study of 60 deceased KT recipients was performed in Vilnius University Hospital Santaros Klinikos between November 2015 and December 2016. Only 37 of them (20 males, 17 females) were eligible for the further investigation. Each participant should have been older than 18 years, without previous history of CVEs (ischemic stroke), cardiovascular events and diseases (myocardial infarction, clinically evident ischemic heart disease, atrial fibrillation) and without clinically evident peripheral artery disease (ankle‐brachial index was >0.9). All 37 KT recipients who met the inclusion criteria were studied before KT, at the day of discharge from hospital and after a median 12 months follow-up. One patient died within first month of observation. All procedures performed in this study were performed with all the subjects’ written informed consent and were in accordance with the ethical standards of the Lithuanian Bioethics Committee and with the 1964 Helsinki declaration and its later amendments.

Our study did not influence the choice of initial or maintenance immunosuppressive treatment. Induction therapy was considered when necessary according to immunological risk (low, medium, or high) and included methylprednisolone, basiliximab, or antithymocyte globulin. All patients received standard maintenance triple therapy which consisted of calcineurin inhibitor (cyclosporin A or tacrolimus), mycophenolate mofetil and methylprednisolone and had stable kidney graft function 6 months after kidney transplant.

### Demographic and clinical data

Demographic and clinical data about age, gender, cause of kidney failure, hypertension, time on dialysis, type of dialysis, donor and recipient cytomegalovirus (CMV) serostatus and prescribed antihypertensive and maintenance immunosuppressive regimen were collected from medical records and interviews with patients. We measured body height and weight and calculated body mass index (BMI) by using formula weight/height^2^ (kg/m^2^). Blood tests were drawn at the time of admission to the hospital for KT, at the time of discharging from hospital after successful KT and after one-year follow-up. They included white blood cell count, hemoglobin, platelet count, creatinine, urea, calcium, ionized calcium, phosphorus, parathormone, albumin, total cholesterol, uric acid, and C-reactive protein (CRP). The estimated glomerular filtration rate (eGFR) was calculated based on the modification of diet in renal disease (MDRD) equation: eGFR_discharge_ – based on creatinine level at the time of discharging from hospital and eGFR_1year_ – based on creatinine 1-year after KT. Data about cardiovascular events within follow-up were also calculated. Cardiovascular events were defined as primary end-point. Bearing in mind, that eGFR is a predictor of long-term kidney graft function, we decided to determine the factors which could forecast the eGFR_1year_.

### Vascular parameters

Before KT, systolic and diastolic blood pressure (BP) were measured by trained medical doctor for three times, with 2 min interval, in supine position after 15 min resting with manual BP monitor (Riester precisa^®^ N Sphygmomanometer, Jungingen, Germany ) on the arm without arterio-venous fistula and average value was recorded. Central BP, mean BP, heart rate, pulse pressure, carotid-femoral pulse wave (cfPWV) velocity and carotid-radial pulse wave velocity (crPWV) were determined using applanation tonometry (SphygmoCor, AtCor Medical Pty Ltd, Sydney, Australia) as previously reported [34]. Direct carotid to radial and carotid to femoral distances were multiplied by 0.8. Only waveforms with operator index greater than 80% were analyzed. We also calculated PWV ratio according to suggested formula by Fortier et al.: PWV ratio = cfPWV/crPWV [35].

### Evaluation of aortic arch calcification

Each patient had their pretransplant posterior to anterior chest X-ray scanned, which were later reviewed by two independent experienced radiologists blinded to patient’s medical data. For evaluation of VC, we used AoAC scale firstly described by Ogawa et al. [30]. This quite simple scale was manually attached to chest X-ray scan and divided aortic arch into 16 slices. Each radiologist counted the calcification affected sectors. After initial evaluation, Cohen's kappa coefficient between experts was 0.71. The mismatching AoAC scores were repeatedly reviewed and resulted in a consensus. The AoAC score values 0 or 1 were considered as no calcification and coded as AoAC (–). Other values were coded as AoAC (+).

### Statistical analysis

Continuous data were expressed as mean ± SD and discrete data as median. Categorical variables were presented as numbers with percentages in parenthesis. For normally distributed continuous data, an *F*-test for testing the equality of two populations before performing Student’s *t*-test was used. Not normally distributed data were analyzed with two-sample Wilcoxon’s test. Chi-square test was applied to categorical variables. Univariate or multivariate linear regressions were used to assess the correlation of eGFR with VC and other factors within follow-up. Univariate Cox regression analysis was conducted to establish the variables associated with cardiovascular events. Due to low event rate, we did not perform a multivariate regression analysis. A two-tailed *p* value <.05 was considered statistically significant. Statistical analysis was performed using R commander (Rcmdr) 3.3.2 version.

## Results

Baseline characteristics of the whole study population as well as according to evidence of AoAC are listed in Table 1.

**Table 1.** Baseline characteristics.

Variables	Mean ± SD
Demographics and comorbid conditions	
Recipient age (years)	46.95 ± 11.96
Recipient gender (men)	20 (54.1%)
BMI (kg/m^2^)	24.04 ± 4.57
Hypertension	32 (86.5%)
Diabetes mellitus	2 (5.4%)
Donor age	46.33 ± 11.26
Donor gender (male)	22 (59.4%)
Hemodynamic and vascular parameters	
Systolic BP (mmHg)	143.78 ± 16.87
Diastolic BP (mmHg)	86.43 ± 12.38
PP (mmHg)	57.38 ± 11.23
Heart rate (beats/min)	72.12 ± 11.47
Central SBP (mmHg)	125.93 ± 15.73
Central DBP (mmHg)	100.21 ± 11.98
cfPWV (m/s)	8.92 ± 2.12
crPWV (m/s)	10.11 ± 1.35
End-systolic BP (mmHg)	124.20 ± 17.51
Biological markers	
CRP (mg/L)	2.49 ± 2.66
LDL cholesterol (mmol/L)	4.27 ± 1.71
HDL cholesterol (mmol/L)	1.24 ± 0.36
Total cholesterol (mmol/L)	5.90 ± 1.21
Albumin (g/L)	44.34 ± 3.48
Calcium (mmol/L)	2.39 ± 0.15
Ionized calcium (mmol/L)	1.14 ± 0.11
Phosphate (mmol/L)	1.67 ± 0.50
PTH (pmol/L)	71.27 ± 57.54
Creatinine	830.38 ± 215.31
Urea	19.16 ± 7.53
Uric acid	292.74 ± 86.22
WBC (×10^9^/L)	6.73 ± 2.00
Hemoglobin (g/L)	120.79 ± 12.56
Platelets (×10^9^/L)	219.35 ± 53.52
Type of dialysis	
HD	30 (81.1%)
PD	7 (18.9%)
Kidney disease duration (years)	14.49 ± 11.51
CMV serology	
CMV donor positive	33 (89.2%)
CMV recipient positive	30 (81.1%)
Drug therapy	
Beta-blockers	24 (70.6%)
CCB	21 (61.8%)
CAAD	21 (61.8%)
Doxazosin	13 (38.2%)
Diuretics	4 (11.8%)
ARBs	3 (8.8%)
Tacrolimus	27 (73.0%)
Cyclosporine	10 (27.0%)

BMI: body mass index; BP: blood pressure; GN: glomerulonephritis; CMV: cytomegalovirus infection; PTH: parathyroid hormone; cfPWV: carotid-femoral pulse wave velocity; crPWV: carotid-radial pulse wave velocity; PP: pulse pressure; PWV: pulse wave velocity; HD: hemodialysis; PD: peritoneal dialysis; eGFR: estimated glomerular filtration rate; LDL: low-density cholesterol; HDL: high-density lipoprotein; CRP: C-reactive protein; RRT: renal replacement therapy; CCB: calcium channel blockers; CAAD: centrally acting antihypertensive drugs; ARBs: angiotensin II receptor blockers.

The mean age of 37 participants was 46.94 ± 11.95 years, two (11.8%) patients had diabetes, 32 (86.49) were with hypertension, 12 (32.43%) with higher than 6.2 mmol/L total cholesterol, seven (18.92%) on peritoneal dialysis and the rest on hemodialysis. Patients without evident calcification were significantly younger (40.94 vs. 52.94 years, *p* = .001), had significantly higher body height (175.15 vs. 167.18 cm, *p* = .028), higher end systolic BP (127.76 vs. 116.23 mmHg, *p* = .038), brachial diastolic and central diastolic BP (91.11 vs. 82.68 mmHg, *p* = .023 and 105.41 vs. 94.00 mmHg, *p* = .005, respectively), higher brachial mean and central mean BP (110.09 vs. 101.25 mmHg, *p* = .029 and 113.76 vs. 102.85 mmHg, *p* = .008, respectively), higher crPWV (10.75 vs. 9.36 m/s, *p* = .002), lower PWV ratio (0.78 vs. 1.02 m/s, *p* = .024). There was no significant difference in time on dialysis before KT between groups.

Table 2 reveals blood test results before KT, on the day of discharging from the hospital and after average 12 months follow-up. Although ionized calcium, phosphorus, parathormone, and eGFR levels did not vary between groups, there was significant difference in calcium concentration and creatinine levels 1-year after transplantation. Patients with AoAC had lower pretransplant hemoglobin level.

**Table 2.** Comparison of blood test results within groups before kidney transplant, on the discharging day and after 1-year follow-up.

	Pretransplant			Based on the discharge data			After 1 year		
	AoAC (−)	AoAC (+)	*p*	AoAC (−)	AoAC (+)	*p*	AoAC (−)	AoAC (+)	*p*
Albumin (g/L)	43.89	44.68	.51	42.72	39.78	.26	42.81	39.9	.33
WBC (10e9/L)	6.36	7.28	.18	8.29	8.05	.83	6.96	9.92	.96
Hemoglobin (g/L)	124.66	115.73	**.03**	105	101.25	.45	134.57	133.83	.9
Platelet (10^9^/L)	208.88	233.33	.2	240.68	233.5	.8	232.64	223.5	.74
Calcium (mmol/L)	2.37	2.41	.42	2.33	2.4	.42	2.33	2.54	**<.001**
Phosphate (mmol/L)	1.59	1.6	.81	0.87	0.77	.66	0.97	1.03	.97
Ca × P (mmol^2^/L^2^)	4.06	3.96	.87	2.02	1.78	.89	2.51	2.51	.97
Ionized calcium (mmol/L)	1.11	1.19	.11	1.22	1.20	.74	1.16	1.20	.22
Parathormone (pmol/L)	57.6	50.35	.76	21.6	27.8	.77	11	12.6	.88
Uric acid (μmol/L)	298.61	280.86	.57	297.5	343	.08	410.69	408	.94
Total cholesterol (mmol/L)	5.81	6.03	.59	6.14	6.44	.47	5.88	5.94	.85
Urea (mmol/L)	19.47	18.06	.6	9.58	10.36	.52	7.87	7.39	.6
Creatinine (µmol/L)	873.72	752.66	.09	155.84	135.75	.37	119.64	88.75	**.007**
eGFR (mL/min/1.73 m^2^)	–	–	–	51.36	54	.75	64.71	80.33	.11

AoAC: aortic arch calcification; eGFR: estimated glomerular filtration rate; Ca × P: calcium phosphate products.

Bold values indicate *p* values < 0.05.

Comparing BP and vascular parameters before and 1-year after KT, significant decrease in brachial systolic BP (143.45 vs. 132.88 mmHg, *p* = .01), central systolic BP (125.93 vs. 119.08, *p* = .01), central diastolic BP (100.21 vs. 89.98 *p* = .007), cfPWV (8.91 vs. 8.05, *p* = .02) was observed. There was no significant difference in brachial or central diastolic BP (86.65 vs. 87.11, *p* = .42) nor in crPWV (10.13 vs. 9.80, *p* = .68) (results not listed in a table).

We analyzed eGFR in 1-year follow-up after successful KT. During first post-transplant year, six (16.21%) patients had worsening KT function. In the univariate linear regression, eGFR_1year_ significantly positively correlated with eGFR_discharge_ and total calcium level based on hospital discharge data (*β* = 0.563, *p* = .004 and *β* = 51.360, *p* = .026, respectively) and negatively with transplant rejection episodes and donor age (*β*=–29.159, *p* = .029 and *β*=–1.469, *p* = .006, respectively). There was no significant correlation with pretransplant PWV ratio or AoAC score. In multivariate linear regression after application of stepwise model selection, two models independently correlated with eGFR_1year_: Model 1 with donor age, donor gender and recipient eGFR_discharge_ and Model 2 with evident AoAC and recipient eGFR_discharge_ (Table 3). When comparing these two models, Model 1 had significantly better descriptive value.

**Table 3.** Factors associated with eGFR_1year_ (univariate and multivariate linear regressions).

			Multivariate			
	Univariate		Model 1		Model 2	
	Standardized coefficients *β*	*p* Value	Standardized coefficients *β*	*p* Value	Standardized coefficients *β*	*p* Value
Recipient age (per 1-year increase)	–0.132	.761	–	–	–	–
Recipient gender (male)	11.929	.235	–	–	–	–
BMI (per 1 m^2^ increase)	0.226	.838	–	–	–	–
Donor gender (male)	17.230	.229	19.882	.05	–	–
Donor age (per 1-year increase)	–1.496	.006	–1.385	.004	–	–
eGFR_discharge_ (mL/min/1.73 m^2^)	0.563	.004	0.349	.05	0.528	.006
Calcium (mmol/L)^a^	51.360	.026	–	–	–	–
Phosphorus (mmol/L)^a^	–9.067	.492	–	–	–	–
Ca × P (mmol^2^/mmol^2^)^a^	–0.358	.95	–	–	–	–
Albumin (g/L)^a^	–0.676	.335	–	–	–	–
Hemoglobin (g/L)^a^	0.585	.114	–	–	–	–
Total cholesterol (mmol/L)^a^	3.892	.308	–	–	–	–
Transplant rejection episodes (yes)	–29.159	.029	–	–	–	–
Time on dialysis (days)	0.001	.776	–	–	–	–
Kidney disease duration (years)	–0.418	.402	–	–	–	–
Calcineurin inhibitor (tacrolimus)	10.588	.317	–	–	–	–
crPWV (m/s)	2.687	.269	–	–	–	–
cfPWV (m/s)	0.666	.873	–	–	–	–
PWV ratio (logarithmed)	12.731	.526	–	–	–	–
Vascular calcification (AoAC (+))	15.619	.116	–	–	12.242	.05

BMI: body mass index; eGFR: estimated glomerular filtration rate; Ca × P: calcium phosphate products; crPWV: carotid-radial pulse wave velocity; cfPWV: carotid-femoral pulse wave velocity; AoAC: aortic arch calcification.

Model 1: *R*-squared 0.65, *p* = .002.

Model 2: *R*-squared 0.35, *p* = .006.

a Blood test results on the discharging day.

In our study, we also collected data about cardiovascular or CVEs in posttransplant period. During one-year follow-up, four (10.81%) participants experienced CVE: two myocardial infarctions, one pulmonary artery embolism, and one stroke. Therefore, the rate of CVE was 7.6 cases per 1000 person-days. Univariate Cox-regression (Table 4) revealed that pretransplant CRP level (HR 1.660, *p* = .007) and PWV ratio (HR 7.549, *p* = .045) predict cardiovascular events. AoAC score had no predictive value (HR 1.044, *p* = .158). Similarly, the other traditional cardiovascular risk factors showed no significant impact on CVE prevalence in study population. We did not perform multivariate Cox-regression analysis due to small sample size and quite low event rate.

**Table 4.** Cardiovascular event risk association with selected variables: univariable Cox regression analysis.

Unadjusted	HR	*p* Value
Age	1.07	.152
Gender (male)	2.89	.357
Diabetes	1.12	.556
Smoking	1.00	.480
Hypertension	2.56	.350
BMI	1.13	.150
Time on dialysis	1.01	.94
CRP	1.660	.007
Total Chol	0.70	.450
LDL-Chol	1.30	.45
P	1.01	.99
Hgb	0.97	.555
AoAC score (≥1)	1.044	.158
MAP	1.02	.589
cfPWV	1.16	.464
rPWV	0.75	.501
PWV ratio	7.549	.045

BMI: body mass index; CRP: C-reactive protein; total Chol: total cholesterol; LDL-Chol: low-density cholesterol; P: phosphate; Hgb: hemoglobin; AoAC: aortic arch calcification; MAP: mean arterial pressure; cfPWV: carotid-femoral pulse wave velocity; crPWV: carotid-radial pulse wave velocity: PWV ratio: pulse wave velocity ratio.

Hazard ratio and 95% confidence interval obtained by univariable and multivariable Cox regression analyses are listed.

## Discussion

It is the first study analyzing pretransplant AoAC not only in the context of cardiovascular/CVEs, but also as predictor of kidney graft function.

The prevalence of VC in kidney transplanted patients is reported to be ranging 24 and 80% [36–38]. In our study, 52.78% of the patients presented AoAC. The cfPWV is considered as the ‘gold standard’ of aortic stiffness measurement and there are many data proving its association with cardiovascular outcomes [39,40]. Previous studies showed no change of aortic PWV in KT patients [41,42], in contrast Zoungas et al. reported amelioration of peripheral PWV 1-year after KT [43]. Moreover, there are few studies considering the PWV ratio as more valuable prognostic factor of mortality than cfPWV [34]. In addition to this, we have found that PWV ratio may predict cardiovascular events. Also, our study has explored the differences of vascular parameters before and 1-year after KT, which have showed the improvement in aortic PWV and BP observed in other similar studies [44].

Comparing to the general population, cardiovascular disease plays more pivotal role in KT recipients, due to annual cardiovascular events risk of 3.5–5% [45]. Despite AoAC being considered as an independent predictor of cardiovascular outcomes [32], our data do not confirm that. In our population, only arterial stiffness was associated with cardiovascular events 1 year after KT. It could be explained by short follow-up, small study population and the exclusion of patients with cardiovascular/cerebrovascular history.

Vascular calcification is pathophysiologically related to inflammation, atherosclerosis, and vascular stiffness, which are usually observed in patients with ESRD [25]. We analyzed only several major risk factors for atherosclerosis before and after KT: hypertension, dyslipidemia, smoking, diabetes, age, inflammation, and CRP levels. Family history of early heart disease was absent in all patients. Besides, VC in large arteries per se is the expression of advanced atherosclerosis. Our study patients with confirmed pretransplant AoAC were older, had hypertension, but no significant difference in inflammatory markers, lipid profile, diabetes, and smoking prevalence.

DeLoach et al. have reported association of CRP with aortic calcification in kidney transplant recipients (KTRs), however, it has not proved by all studies [29,46]. Our findings show that pretransplant CRP may predict cardiovascular events which are in line with previous study [47].

Abnormal bone and mineral metabolism tends to persist in most KTRs, even though a successful KT improves regulatory hormones levels and excretion of accumulated waste products and electrolytes [48]. The prevalence of hypercalcemia in kidney transplant patients is estimated up to 66% [49], though the methods to determine it vary. Hypercalcemia and hypophosphatemia are resulted by parathormone induced calcium re-absorption and phosphorus excretion [50,51]. In our study, we observed that total serum calcium, but not ionized calcium, levels 1-year after KT were higher in patients with AoAC. It is known that hypercalcemia could be related to decline of transplanted kidney function in two ways: vasoconstriction and calcium deposition in tubulo-interstitium [52–54]. Some studies suggest that hypercalcemia after KT could be caused by reabsorption of VC [55]. Moreover, high calcium concentration in blood and urine can impair sodium and water reabsorption leading to the compensatory enhancement of eGFR and polyuria. Our data show that 1-year after KT patients with AoAC have significantly higher calcium levels and nonsignificantly but higher eGFR at the same time. Our results suggest that pretransplant AoAC score after adjustment for recipient eGFR_discharge_ independently correlates with recipient eGFR_1year_, but has worse descriptive value than recipient eGFR_discharge_ combined with donor age and gender. Unfortunately, we did not measure urine calcium level and had no information about urine volume changes in our patients. Further research is needed to define the predictive role of AoAC progression to eGFR change and prognosis.

The limitations of this current research include small size of study population, which together with low number of cardiovascular events are related to weak statistical power in predicting the impact of AoAC and arterial stiffness on cardiovascular outcomes. This may be explained by enrollment of participants without previous history of cardiovascular/CVEs. On the other hand, it may represent better understanding of KT role in VC and its related outcomes. Future studies with larger sample sizes would be necessary to determine the interrelationship between AoAC, measured by more accessible in clinical practice and cost-effective X-ray, kidney function and cardiovascular events after KT with longer observation period.

In conclusion, AoAC is prevalent among kidney transplanted patients, but there is lack of evidence that pretransplant AoAC could predict early cardiovascular outcomes and decline of graft function in patients without previous history of cardiovascular/CVEs. Arterial stiffness and inflammation markers might be considered to have predictive value, but more data are needed for further risk stratification in renal transplant recipients.
